# Angiotensin‐(1‐7) inhibits sodium transport via Mas receptor by increasing nitric oxide production in thick ascending limb

**DOI:** 10.14814/phy2.14015

**Published:** 2019-03-06

**Authors:** Paula Dibo, Rodrigo O. Marañón, Kiran Chandrashekar, Fernando Mazzuferi, Guillermo B. Silva, Luis A. Juncos, Luis I. Juncos

**Affiliations:** ^1^ Department of Basic Research J. Robert Cade Foundation Cordoba Argentina; ^2^ Department of Medicine/Nephrology University of Mississippi Medical Center Jackson Mississippi; ^3^ Department of Cell and Molecular Biology University of Mississippi Medical Center Jackson Mississippi; ^4^ Department of Medicine/Nephrology Central Arkansas Veterans Healthcare System University of Arkansas for Medical Sciences Little Rock Arkansas; ^5^ Gabinete de Tecnología Médica (GATEME‐UNSJ) Universidad Nacional de San Juan ‐ Consejo Nacional de Investigaciones Científicas y Técnicas – CONICET San Juan Argentina

**Keywords:** Angiotensin 1‐7, hypertension, mas receptor, TAL

## Abstract

Sodium transport in the thick ascending loop of Henle (TAL) is tightly regulated by numerous factors, especially angiotensin II (Ang II), a key end‐product of the renin‐angiotensin system (RAS). However, an alternative end‐product of the RAS, angiotensin‐(1‐7) [Ang‐(1‐7)], may counter some of the Ang II actions. Indeed, it causes vasodilation and promotes natriuresis through its effects in the proximal and distal tubule. However, its effects on the TAL are unknown. Because the TAL expresses the Mas receptor, an Ang‐(1‐7) ligand, which in turn may increase NO and inhibit Na+ transport, we hypothesized that Ang‐(1‐7) inhibits Na transport in the TAL, via a Mas receptor/NO‐dependent mechanism. We tested this by measuring transport‐dependent oxygen consumption (VO_2_) in TAL suspensions. Administering Ang‐(1‐7) decreased VO_2_; an effect prevented by dimethyl amiloride and furosemide, signifying that Ang‐(1‐7) inhibits transport‐dependent VO_2_ in TAL. Ang‐(1‐7) also increased NO levels, known inhibitors of Na+ transport in the TAL. The effects of Ang‐(1‐7) on VO_2_, as well as on NO levels, were ameliorated by the Mas receptor antagonist, D‐Ala, in effect suggesting that Ang‐(1‐7) may inhibit transport‐dependent VO_2_ in TAL via Mas receptor‐dependent activation of the NO pathway. Indeed, blocking NO synthesis with L‐NAME prevented the inhibitory actions of Ang‐(1‐7) on VO_2_. Our data suggest that Ang‐(1‐7) may modulate TAL Na+ transport via Mas receptor‐dependent increases in NO leading to the inhibition of transport activity.

## Introduction

The renin‐angiotensin system (RAS) cascade is a key modulator of cardiovascular and renal function predominantly via the actions of angiotensin II (Ang II). However, the discovery of additional biologically active RAS end‐products has changed the paradigm of RAS signaling. These alternative end‐products are the result of distinctive cleavage pathways. While several angiotensin‐related peptides have been described, of particular interest is the heptapeptide angiotensin 1‐7 [Ang‐(1‐7)], which is formed via the catalytic action of angiotensin‐converting enzyme 2 (ACE2) directly on angiotensin II or indirectly on angiotensin I (Wysocki et al. 2008). This ACE2/Ang‐(1‐7) axis may serve as an important counter‐regulator to the physiologic and pathophysiologic actions of angiotensin II, particularly in the kidney, where Ang II is a primary regulator of many aspects of renal function, including the renal microcirculation, glomerular filtration, tubular transport, and renal growth (Hebert and Andreoli 1984; Ortiz et al. 2001; Wysocki et al. 2008). Indeed, there are several lines of evidence suggesting that Ang‐(1‐7) plays an important role in the kidney. First, the enzymes required for its biosynthesis and degradation are abundant in the kidney (Gurley et al. 2006; Chappell 2016). Moreover, an additional pathway exists for its biosynthesis in the kidney via the metalloendopeptidase neprilysin, which forms Ang‐(1‐7) directly from Ang I or Ang‐(1‐12) independent of ACE2 (Chappell 2016). Second, Ang‐(1‐7) has been found to potentially regulate salt and water balance, as well as blood pressure; it causes vasodilation of rabbit afferent arterioles (Ren et al. 2002) and induces marked natriuresis in normotensive rats and dogs (Handa et al. 1996; Heller et al. 2000), whereas inhibiting Ang‐(1‐7) or blocking its receptor (Mas), induces an increase in blood pressure and blunts the effects of ACE inhibitors (Ferrario et al. 2005). Despite these biologic actions of Ang‐(1‐7) in the kidney, the precise mechanisms are incompletely understood. In particular, little is known whether Ang‐(1‐7) exerts biologic effects in the thick ascending loop of Henle (TAL).

The TAL plays an essential role in modulating Na+ balance, and consequently long‐term blood pressure (Jung et al. 2011). In order to maintain appropriate Na+ homeostasis during various physiologic stresses, Na+ transport in the TAL must be tightly regulated *via* the coordinated actions of various factors, in particular, the counterregulatory effects of Ang II and nitric oxide (NO) (Hebert and Andreoli 1984; Greger 1985, 2000; Ortiz et al. 2001). Because Ang‐(1‐7) may exert some of its biologic actions in other tissues via Mas receptor‐mediated NO production, and the Mas receptor is abundant in the kidney medulla (Gwathmey‐Williams et al. 2010), we hypothesized that Ang‐(1‐7) couples to TAL Mas receptors, causing NO levels to increase, with a resultant decrease in Na+ transport. Our results suggest that Ang‐(1‐7) is a novel modulator of Na+ transport in the TAL. Because its effects on transport‐dependent oxygen consumption (VO_2_) and/or the NO pathway are blunted by a Mas receptor or NO synthesis blockers, Ang‐(1‐7) appears to be exerting its effects on TALs via Mas receptor‐mediated increases in NO production.

## Methods

### Ethical approval

All animal experiments were performed with the approval of the Institutional Animal Care and Use Committee of the J. Robert Cade Foundation (#3‐2016) and conducted according to the National Institute of Health Guide for the Care and Use of Laboratory Animals and conform to the principles and regulations of Experimental Physiology, as described by Grundy (2015) (Grundy 2015). After harvesting the renal tissue, the rats were euthanized under deep anesthesia with an overdose of isoflurane followed by exsanguination/cardiac extraction.

### Experimental animals

Young male Wistar rats weighing 150–200 g were bred and maintained in a closed rat colony at the J. Robert Cade Foundation. They were exposed to light‐dark cycles of 12 h each with ad libitum access to standard chow diet (Grupo Pilar, Córdoba, Argentina) and tap water for 7–10 days before the experiments. On the day of the experiment, animals were anesthetized with isoflurane, the renal tissues harvested, and the animals euthanized as described above.

### Medullary TAL suspensions

Medullary TAL suspensions were prepared as previously described (Chamberlin et al. 1984; Ortiz et al. 2001; Silva and Garvin 2009a). Briefly, the kidneys were perfused via the abdominal aorta with 40 mL of HEPES‐buffered physiological saline, then removed, cut in coronal slices from which the inner stripe of the outer medulla was dissected. The tissue was minced and incubated at 37°C for 30 min in 0.1% collagenase type I, while being oxygenated with 100% oxygen and gently shaken in 5‐min intervals. The resulting tubule suspension was filtered using a 250‐*μ*m nylon mesh and centrifuged again for 2 min. The pellet was rinsed and resuspended in 1 mL cold HEPES‐buffered physiological saline.

### Measurement of transport‐related oxygen consumption

We examined whether Ang‐(1‐7) inhibits TAL transport by measuring its effects on transport‐dependent VO_2_ (which correlates with actual transport), as previously described (Mandel 1986; Ortiz et al. 2001; Silva and Garvin 2008, 2009b). For this, TAL cells were suspended in 0.1 mL of physiological saline warmed to 37°C and equilibrated with 100% oxygen. They were placed in a closed chamber at a 37°C temperature, while VO_2_ was continuously recorded using a Clark electrode. After obtaining a basal slope, the desired treatment agent(s) were added (e.g., Ang1‐7, furosemide, d‐ALA, L‐NAME, etc.). The effect of the treatment was measured after stabilization of the new slope (>4 min). All experiments were completed within 18–20 min. Data were digitalized and slopes were calculated using MATLAB v.12, (Mathworks, MA). The results were expressed as percent inhibition from basal levels.

### Measurement of intracellular NO

Intracellular NO in TAL cells was measured using 4, 5‐diaminofluorescein diacetate (DAF‐2), a NO‐selective fluorescent dye. After loading TAL cells with DAF‐2, a 10‐min equilibration period was allowed. Measurements were then taken for 10 sec every minute for 5 min to ascertain basal NO levels. Ang‐(1‐7) was then added to the bath, after a 5‐min equilibration period, measurements were obtained as before for a further 5 min. To determine whether Ang‐(1‐7) was exerting its effect via the Mas receptor, experiments were performed in the presence of D‐Ala^7^‐Ang‐(1‐7), a Mas receptor blocker, which was added to the chamber at the beginning of the equilibration period. To confirm that the changes in VO_2_ were due to transport‐related VO_2_, additional experiments were performed in the presence of furosemide and dimethyl amiloride. Time control experiments were also performed to test the stability of the fluorescent dye.

### Statistical analysis

Data are reported as means (±SD). Differences in means were analyzed using either Student's *t*‐test for paired experiments or an unpaired *t*‐test, applying Hochberg's adjustment when appropriate to determine significance.

## Results

### The effect of Ang‐(1‐7)‐induced Mas receptor activation on transport‐dependent VO_2_ in TAL

**Figure 1:**
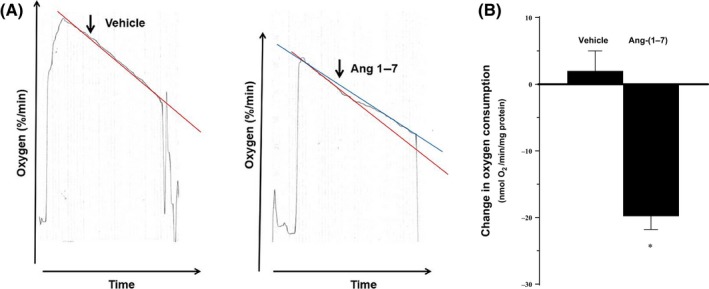
Effect of Ang‐(1‐7) on VO_2_ in TAL. Change in VO_2_ caused by Ang‐(1‐7). (Vehicle: *n* = 5; Ang‐(1‐7): 0.1 nmol/L: *n* = 5; 1 nmol/L: *n* = 5). (A) shows tracing of representative experiments, while (B) is a graphical summarization of the results.

We first examined the effect of Ang‐(1‐7) on basal VO_2_. As depicted in Fig. 1A, in the absence of Ang‐(1‐7), VO_2_ was linear over the entire time course of the experiments (*r* = 0.948 ± 0.015). Administration of 0.1–1 nmol/L of Ang‐(1‐7) consistently decreased VO_2_. The maximal effect was observed at the 1 nmol/L dose (it inhibited VO_2_: Vehicle: 2 ± 3 vs. Ang‐(1‐7): −19.2 ± 2 mmolO_2_/min/mg protein; Fig. 1B). Consequently, all subsequent experiments were performed using that dose.

**Figure 2:**
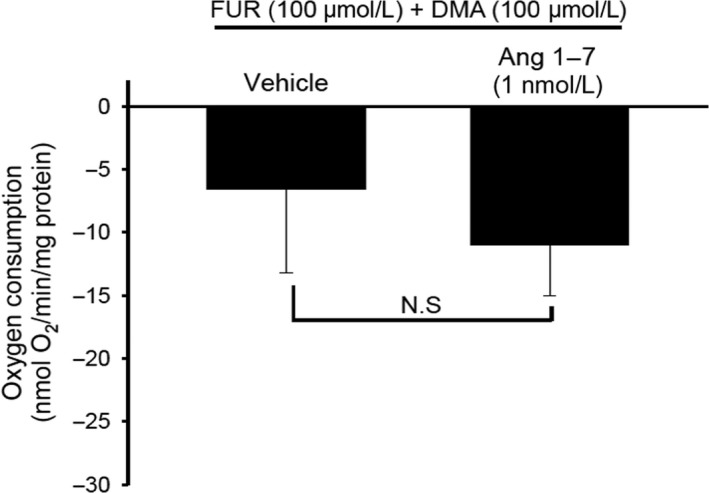
Effect of Ang‐(1‐7) on VO_2_ in TAL during the inhibition of NHE exchanger and the NKCC2‐cotransporter. Addition of Ang‐(1‐7) (1 nmol/L) in the presence of apical transporters inhibitors NHE exchanger and the NKCC2‐cotransporter inhibitors dimethyl amiloride (DMA, 100 *μ*mol/L) and furosemide (FUR, 100 *μ*mol/L) did not inhibit VO_2_, indicating that the effects of Ang‐(1‐7) are related to transport (*n* = 5).

To ensure that the fall in VO_2_ induced by Ang‐(1‐7) reflected decreased transport, we tested whether simultaneous inhibition of NKCC2 and NHE (the two predominant transporters in the TAL) abolished Ang‐(1‐7)'s effect on VO_2_. Pretreatment of TAL suspensions with 100 *μ*mol/L each of furosemide and dimethyl amiloride (DMA), decreased the mean VO_2_ by 6.6 ± 5.9%. Adding Ang‐(1‐7) to this dual cocktail did not further change VO_2_ (11.5 ± 4.1%; Fig. 2), demonstrating that intact NKCC2 and/or NHE transporters are necessary for Ang‐(1‐7) to inhibit VO_2_ in TAL cells. Thus, our results indicate that the effect of Ang‐(1‐7) on VO_2_ is likely secondary to the inhibition of transport.

**Figure 3:**
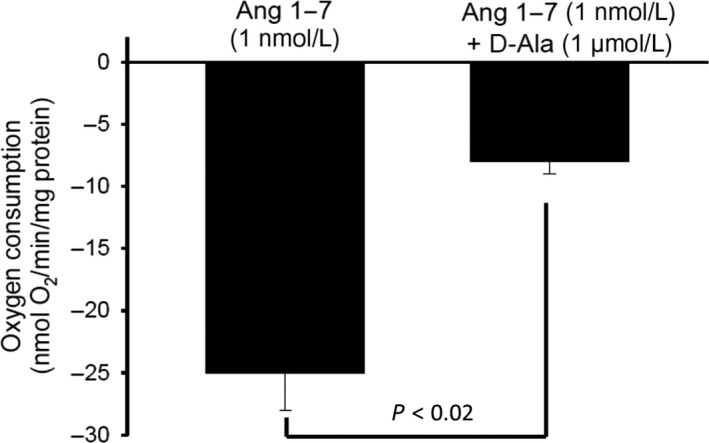
Effect of Ang‐(1‐7) on TAL oxygen consumption during inhibition of Mas receptors. Addition of Ang‐(1‐7) (1 nmol/L) in the presence of the Mas receptor antagonist D‐Ala7‐Ang‐(1‐7) (1 *μ*mol/L), did not inhibit TAL oxygen consumption (*n* = 6).

In the last experiments of this series, we established whether Ang‐(1‐7) exerted its effects via Mas receptors by using a specific Mas receptor antagonist. D‐Ala^7^‐Ang‐(1‐7) prevented the inhibitory effect of Ang‐(1‐7) on VO_2_; Ang‐(1‐7) inhibited VO_2_ by 23.5 ± 1.64% vs. 8 ± 1.7% in the absence and presence of D‐Ala^7^‐Ang‐(1‐7), respectively (Fig. 3). Thus, these first series of experiments provide substantial evidence that Ang‐(1‐7) activates Mas receptors in the TAL which results in a decrease in transport‐dependent VO_2_.

### The role of NO on Ang‐(1‐7) effects on TAL

**Figure 4:**
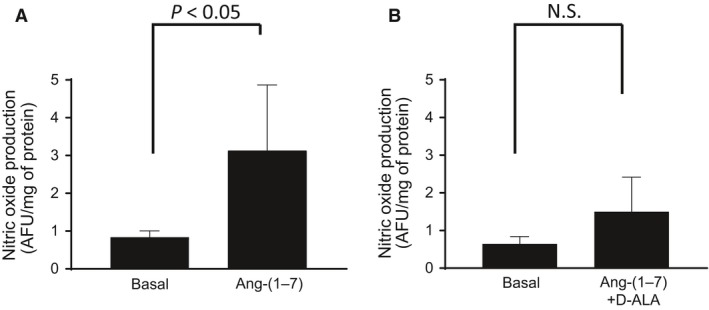
Change in TAL nitric oxide production by Ang‐(1‐7) and during inhibition of the Mas receptor. While the addition of Ang‐(1‐7) (1 nmol/L) stimulated nitric oxide production (A, *n* = 5), the addition of Ang‐(1‐7) (1 nmol/L) in the presence of the Mas receptor antagonist D‐Ala7‐Ang‐(1‐7) (1 *μ*mol/L), did not stimulate nitric oxide production (B, *n* = 5).

In the second series of experiments, we evaluated the role of NO in mediating the effects of Ang‐(1‐7) on transport‐dependent oxygen consumption in TAL. We first tested whether Ang‐(1‐7) increases NO levels in TAL suspensions in a Mas receptor‐dependent manner. For this, we measured NO levels in TAL suspensions exposed to Ang‐(1‐7), in the presence and absence of the Mas receptor antagonist. Ang‐(1‐7) increased NO levels in TAL cells (from 0.83 ± 0.14 to 3.22 ± 1.52 Arbitrary Fluorescence Units (A.F.U./mg of protein; Fig. 4A), indicating that Ang‐(1‐7) increases NO production in the TAL. Addition of the Mas receptor antagonist to the cells substantially reduced Ang‐(1‐7)‐induced generation of NO (Fig. 4B).

**Figure 5:**
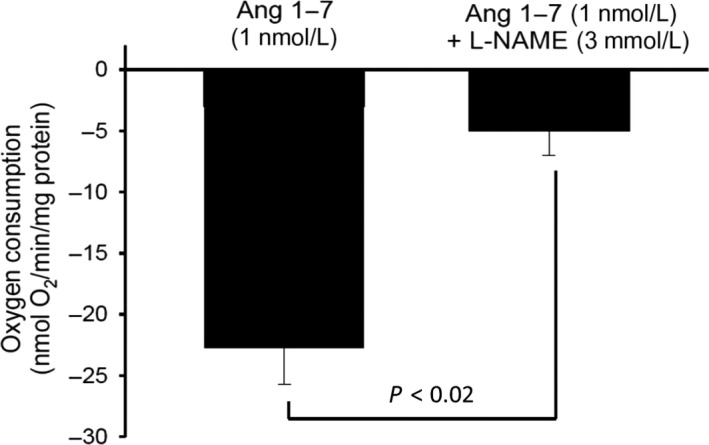
Effect of Ang‐(1‐7) on TAL oxygen consumption during inhibition of nitric oxide synthase (NOS). Addition of Ang‐(1‐7) (1 nmol/L) in the presence of the NOS inhibitor L‐NAME (3 mmol/L, 8 min incubation), did not inhibit TAL oxygen consumption (*n* = 5).

Finally, we determined whether the increases in NO we observed in response to Ang‐(1‐7) were sufficient to account for the decreases in transport‐dependent VO_2_. For this, we tested whether blocking NO generation with N*ω*‐Nitro‐l‐arginine methyl ester (L‐NAME) prevents ANG‐(1‐7)‐induced decreases in VO_2_. We found that Ang‐(1‐7) decreased oxygen consumption by 22% in the absence of L‐NAME, but by only 2% in its presence (Fig. 5), indicating that increases in NO generation may be responsible for Ang‐(1‐7)‐induced inhibition of transport‐dependent VO_2_ in the TAL.

## Discussion

Angiotensin II has powerful vasopressor and antinatriuretic actions. Increasing evidence suggests that some of its alternative metabolites, in particular Ang‐(1‐7), have opposing actions which may provide an important counter‐modulating effect to angiotensin II. Our current study, evaluated one of the mechanisms by which Ang‐(1‐7) may moderate the effects of angiotensin II on the TAL (an important target for its actions). We found that Ang‐(1‐7) decreased transport‐dependent VO_2_ in TAL cells. This inhibitory effect of Ang‐(1‐7) was associated with increased PI3K activity and NO levels. Moreover, blocking NO prevented the effect of Ang‐(1‐7) on VO_2_ indicating NO‐dependence of Ang‐(1‐7)'s effects on the TAL. Finally, all of the above effects of Ang‐(1‐7) could be prevented by blocking the Mas receptor.

In contrast to angiotensin II, Ang‐(1‐7) has natriuretic effects. DelliPizzi et al., found that a low dose of Ang‐(1‐7) (0.1 pmol/mL), induces natriuresis in isolated rat kidneys with preserved tubular function (da Silva et al. 2006). By using this preparation, they excluded the systemic influences of Ang‐(1‐7) thus providing strong evidence for a local effect. However, the segments where it predominantly exerts its actions was unclear. Its well‐known vasodilator effects on the renal vasculature may facilitate sodium excretion, but it is unlikely that this played a role in a preparation that is already characterized by profound baseline vasodilation. A tubular effect seemed likely, a notion supported by studies showing that Ang‐(1‐7) is present in several nephron segments. Indeed, there have been several in vitro and in vivo studies showing that Ang‐(1‐7) directly effects tubular transport (Garcia and Garvin 1994; Handa et al. 1996; da Silva et al. 2006; Castelo‐Branco et al. 2012; Chappell et al. 2014). For instance, Handa et al., found that Ang‐(1‐7) inhibited transport‐dependent VO_2_ in in vivo and in isolated proximal convoluted tubules (7). Ang‐(1‐7) was also found to modulate NHE3 and [Ca^2+^] in proximal tubules in vivo (Castelo‐Branco et al. 2012), and to have a biphasic effect on transport‐dependent VO_2_ in isolated perfused S2 segments of the proximal tubule (Garcia and Garvin 1994).

Despite this abundance of data regarding the actions of Ang‐(1‐7) in the proximal tubule, its role in the TAL segment is not known. Because of the importance of the TAL in regulating sodium and water homeostasis, we examined the effect of Ang‐(1‐7) in the TAL. The thick ascending loop of Henle actively reabsorbs up to 30% of the filtered Na^+^, predominantly through a Na^+^/K^+^/2Cl^−^ mechanism (Chamberlin et al. 1984), which is tightly regulated by a variety of factors, including angiotensin II, endothelin, prostaglandins, and NO (Herrera et al. 2006, 2009). Our study adds Ang‐(1‐7) to this list of regulating factors; we found that it has a considerable effect on transport‐dependent VO_2_ in the TAL suspensions. This raises the possibility that Ang‐(1‐7) in the renal medulla may regulate Na+ transport in TAL and thus potentially play an important role in regulating salt and water balance. Further studies will be necessary to quantify the impact of its effects in diverse conditions in vivo, and whether modulation of its actions proves to be an effective therapeutic option.

We next sought out to uncover the key downstream signals by which Ang‐(1‐7) is exerting its effects on the TAL. One of the mechanisms by which Ang‐(1‐7) exerts its effects in other cells and tissues is via binding to a G‐coupled protein receptor called the Mas receptor. Because this receptor is known to be expressed in the kidney (Pinheiro and Simões E Silva 2012) and especially in the renal medulla (Angus et al. 1986), we tested whether the Mas receptor mediated the effect of Ang‐(1‐7) on TAL VO_2_. We found that blocking the Mas receptor not only inhibited Ang‐(1‐7) effects on VO_2_, but also Ang‐(1‐7)‐induced increases in NO, suggesting that Ang‐(1‐7) may inhibit transport in the TAL via Mas receptor‐dependent activation of the NO pathway. NO is considered to be one of the major factors that determine transport along various nephron segments. The balance between NO, angiotensin II and oxidative stress is thought to largely determine transport activity in the TAL. While all three NO synthase isoforms are expressed in the TAL, NOS3 is reported to be the major source and consequently have the most significant role in regulating TAL transport (Herrera et al. 2006). Moreover, the NOS3 present in the TAL is regulated by the same factors that stimulate its activity in endothelial cells (Angus et al. 1986; Korenaga et al. 1994; Hirata et al. 1995). Ang‐(1‐7)‐mediated Mas receptor activation is one such factor that induces phosphorylation of NOS3, and thus results in increased NO generation (Heitsch et al. 2001; Wiemer et al. 2002; Sampaio et al. 2007; Samuel et al. 2012). Our studies found that this reaction also takes place in TAL; Ang‐(1‐7)‐induced increases in NO production in the TAL was absent in the setting of Mas receptor blockade. Our final experiments demonstrated that Ang‐(1‐7)'s ability to decrease transport‐dependent VO_2_ was dependent on NO generation. Indeed, blocking NO generation with L‐NAME prevented Ang‐(1‐7)‐induced inhibition of oxygen consumption. These data indicate that Ang‐(1‐7) increases nitric oxide production in the TAL via activation of the Mas receptor, resulting in Ang‐(1‐7)‐induced inhibition of TAL transport‐related VO_2_. It is interesting to note that this mechanism appears to also be present in the proximal tubule; Su et al. (2017) reported that Ang‐(1‐7) inhibits Na+ transport in proximal tubule cells via Mas receptor‐dependent NO production. Thus, it appears likely that Ang‐(1‐7) counters the effects of angiotensin II in various renal segments including the vasculature, proximal tubule, and TAL.

### Study limitations

The main limitation of our study is that despite its substantial effect on transport‐dependent VO_2_ in TAL suspensions, we did not quantify its effect in vivo. This may be particularly difficult because of its systemic effects, as well as its effects on the distinct renal segments. We also did not elucidate whether its primary effect is via the NKCC2 or NHE3 transporters. These studies will be necessary to further elucidate its role in distinct pathophysiologic conditions, and to determine whether it may have a potential role in hypertension, heart failure or other conditions where diuresis may be advantageous. Finally, we only evaluated the role of NO; other pathways, and interactions may be as important in determining the net effect of Ang‐(1‐7) on TAL, and in total sodium homeostasis in vivo. Further studies are needed to evaluate these possibilities.

## Conclusions

Our results show that Ang‐(1‐7) increases NO production in the TAL via activation of Mas receptors, thereby resulting in Ang‐(1‐7)‐induced inhibition of TAL transport‐related VO_2_. Our results raise the possibility that Ang‐(1‐7) may play an important role in modulating sodium transport in the TAL and consequently natriuresis. Thus, novel therapies focused on modulating Ang‐(1‐7), its receptor, or signaling pathway, could potentially be beneficial in conditions in which increased excretion may be desired such as salt‐sensitive hypertension (Aviv et al. 2004), or heart failure (Ellison 1984).

## Conflict of Interest

The authors have no conflicts of interest to declare.
